# Undifferentiated Pleomorphic Sarcoma: A Case Report

**DOI:** 10.7759/cureus.73422

**Published:** 2024-11-11

**Authors:** Nivetha Munuswamy, Madan Sundar, Kuberan Krishnan, Magesh Chandran, Karpagam R K

**Affiliations:** 1 Department of General Surgery, Sree Balaji Medical College & Hospital, Chennai, IND; 2 Department of Radiology, Saveetha Medical College and Hospital, Saveetha Institute of Medical and Technical Sciences (SIMATS) Saveetha University, Chennai, IND

**Keywords:** immunohistochemistry, limb-salvage surgery, mesenchymal tumor, postoperative radiotherapy, thigh mass, undifferentiated pleomorphic sarcoma (ups)

## Abstract

Undifferentiated pleomorphic sarcoma (UPS) is an uncommon and aggressive soft tissue tumor primarily affecting older adults, often presenting as a rapidly enlarging, painless mass in the extremities or other deep-seated locations. This case involves a 55-year-old woman who experienced a rapidly progressive and painful swelling in her right thigh following trauma. Imaging studies identified a large, lobulated tumor in the adductor muscles, close to the femur and pubic ramus, with no bone involvement or evidence of distant metastasis. The patient underwent surgical excision, and histopathological analysis confirmed high-grade UPS, necessitating adjuvant radiotherapy due to narrow surgical margins. Follow-up evaluations, including MRI scans at six months and one year, revealed no signs of disease recurrence. The case underscores the critical role of early detection, multidisciplinary treatment planning, and rigorous postoperative monitoring to improve outcomes and reduce the risk of recurrence in patients with UPS.

## Introduction

Soft tissue sarcomas are malignant tumors of mesenchymal origin, accounting for approximately 1-2% of all malignancies and subdivided into over 50 histological subtypes [1]. Among these, undifferentiated pleomorphic sarcoma (UPS), previously referred to as malignant fibrous histiocytoma (MFH), is one of the most aggressive and poorly differentiated subtypes [2]. UPS represents about 5% of all soft tissue sarcomas and is characterized by a high degree of cellular pleomorphism, with no distinct line of differentiation [3]. Due to its undifferentiated nature, UPS is often diagnosed only after ruling out other sarcoma subtypes.

The incidence of UPS increases with age, with the highest risk observed after the seventh decade of life, and it predominantly affects males [4]. Exposure to radiation, especially as part of prior cancer treatments, increases the likelihood of developing UPS, often in the irradiated area years later. Additionally, certain genetic conditions, such as Li-Fraumeni syndrome, Neurofibromatosis type 1, and Retinoblastoma, predispose individuals to sarcomas, including UPS, due to inherited gene mutations. Chronic inflammation or injury, including from ulcers, burns, or long-standing wounds, may also contribute, as persistent tissue damage can lead to cellular changes and mutations. A history of other cancers, particularly soft tissue or bone cancers, further raises the risk, possibly due to genetic susceptibility or past treatments. Finally, environmental carcinogens, like phenoxyacetic acid in herbicides and chlorophenols in wood preservatives, may play a role in sarcoma development, though evidence for this is still limited [4].

These tumors can develop in any part of the body; however, they are most commonly found in the lower extremities, retroperitoneum, arms, and, less frequently, in the head and neck region [5,6]. Clinically, UPS presents as a rapidly enlarging, painless mass, although symptoms can vary depending on the tumor’s location and size. Due to its aggressive nature, UPS is prone to local recurrence and distant metastasis, most commonly to the lungs.

Despite advancements in imaging and histopathology, the prognosis for UPS remains poor, particularly for large, deep-seated tumors or those diagnosed at an advanced stage [6]. Standard treatment typically involves wide surgical excision, with or without adjuvant radiotherapy and chemotherapy; however, recurrence rates remain high, underscoring the need for continued research and improved therapeutic strategies [6,7]. Although trauma causing the development of soft tissue sarcoma is rare, a few cases have been reported in recent decades [7]. Given its rarity and clinical challenges, case reports like this provide valuable insights into the management and outcomes of patients with UPS.

## Case presentation

A 55-year-old female, previously in good health, presented to the outpatient department of general surgery with a painful mass on the medial aspect of her right thigh, following minor trauma. The minor trauma occurred six months before the patient noticed the swelling, which appeared about one month before her presentation to the clinic with a five-month interval between the trauma and the development of the swelling. There was no indication of any other risk factors mentioned for this patient, such as weight loss or appetite loss. Additionally, there was no mention of any prior radiation exposure, genetic conditions, chronic inflammation, or environmental carcinogen exposure that would typically contribute to the risk of developing a condition like Undifferentiated Pleomorphic Sarcoma (UPS).

She first noticed the swelling one month prior, and it had progressively increased in size. The patient denied any associated symptoms, such as paraesthesia, numbness, weight loss, or loss of appetite. Her vital signs at the time of presentation were unremarkable.

**Figure 1:**
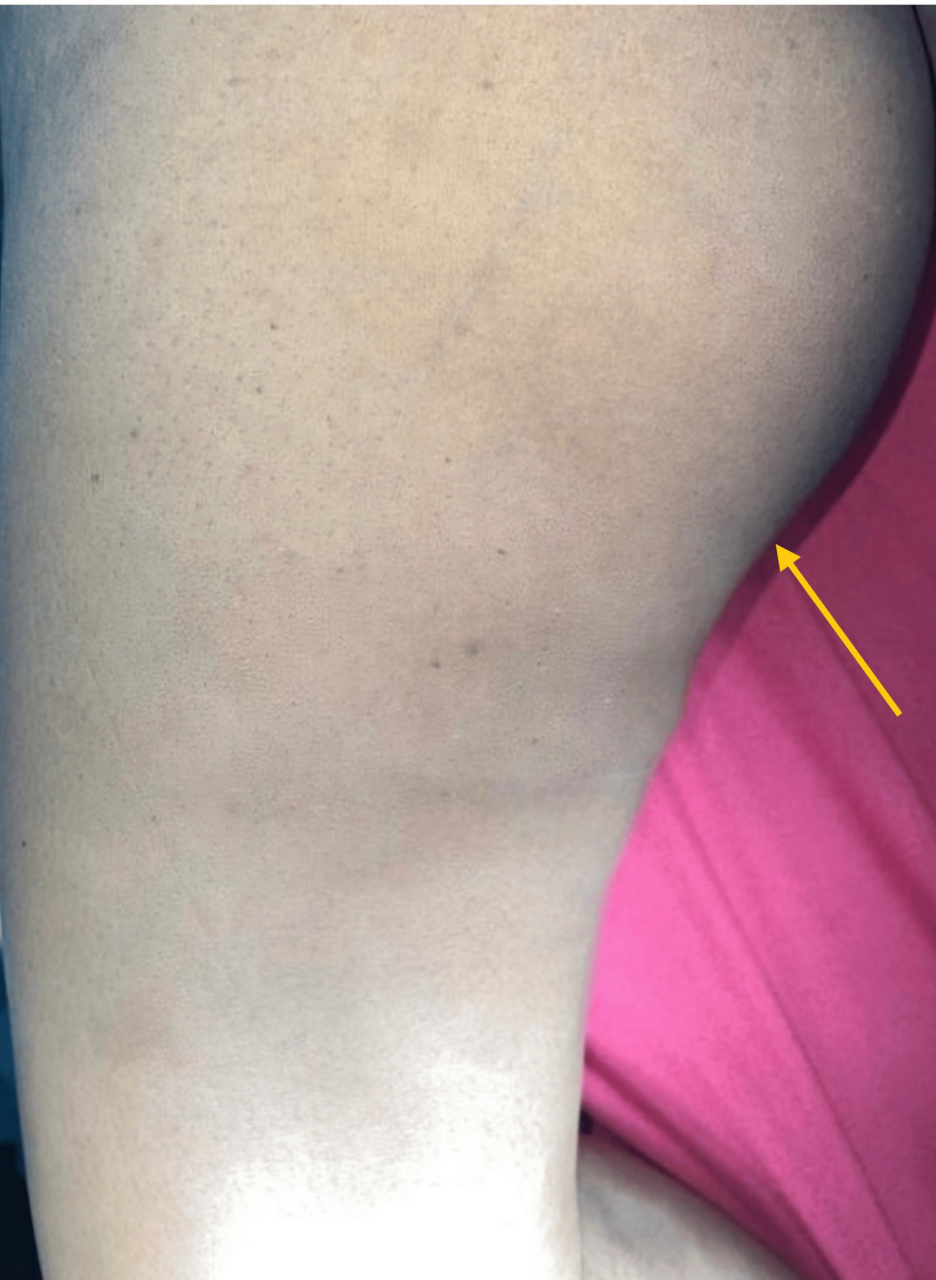
Clinical image of the right thigh showing swelling of size 30 x 20 cm over the medial aspect. yellow arrow - depicting the lesion

During the physical examination, the patient was able to walk without difficulty. A diffuse swelling (yellow arrow), approximately 30 x 20 cm in size, was observed, occupying the medial compartment of the right thigh entirely (Figure 1). Notably, there was only a single swelling present, and the skin over it appeared normal, without any signs of inflammation. Upon palpation, the swelling felt hard and had a smooth surface, with no evidence of fluctuation, transillumination, or pulsation. The swelling exhibited restricted mobility in all directions, and there were no signs of inguinal lymphadenopathy. Sensation in the affected area was intact.

Based on the clinical examination findings, there was a suspicion of a soft tissue tumor on the medial side of the right thigh. Given the combination of a growing mass and potential skeletal complications, a thorough diagnostic evaluation was deemed necessary to determine the nature of the mass and the most appropriate management approach, considering the patient's overall health and the absence of other significant medical or family risk factors.

**Figure 2:**
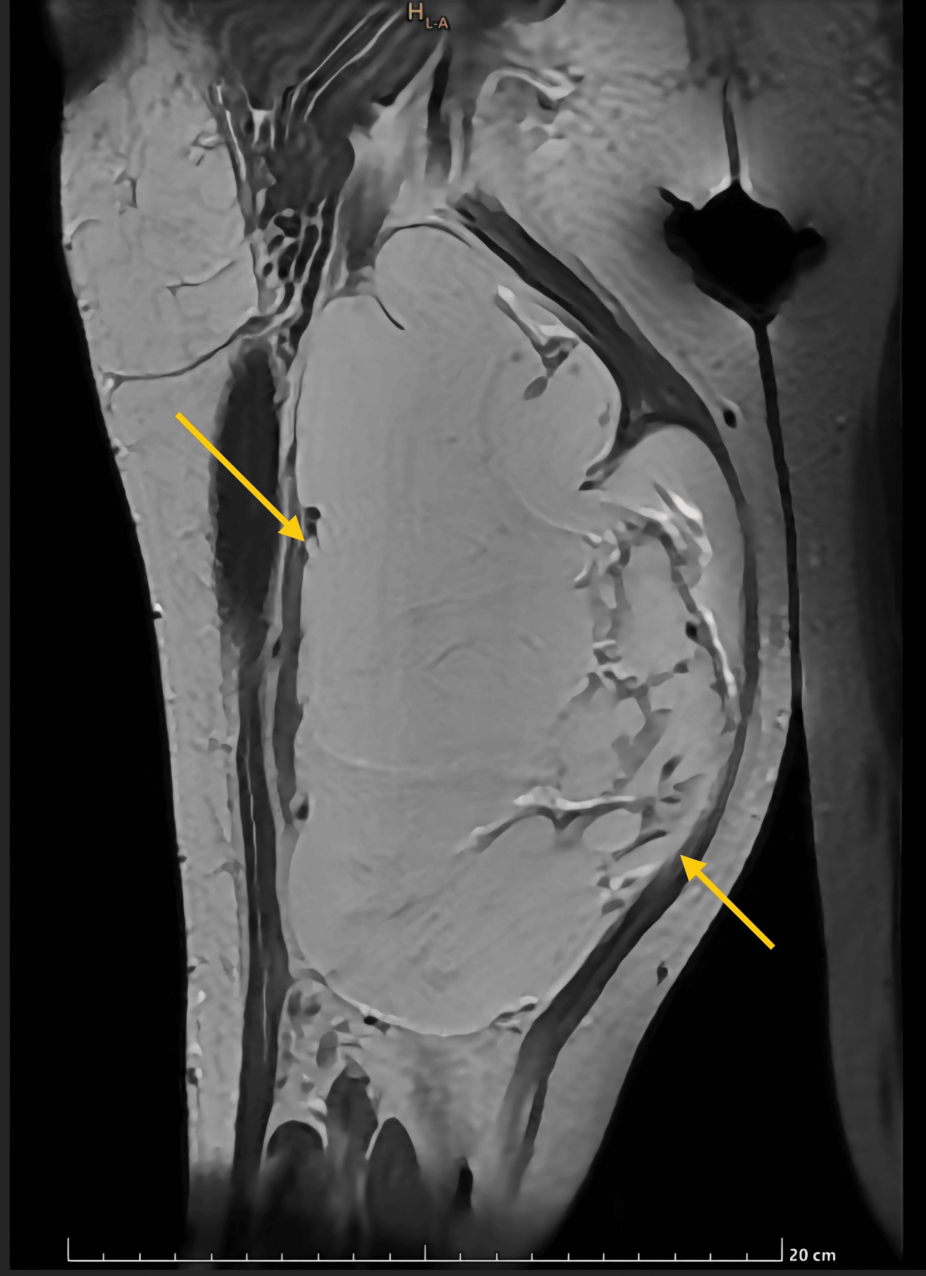
Coronal section of MRI of the right thigh showing the lesion involving the adductor muscles. yellow arrows - depicting the lesion

Standard blood and urine investigations yielded unremarkable results. Subsequently, the patient was advised to undergo magnetic resonance imaging (MRI) of the right thigh, which revealed a relatively well-defined, lobulated, heterogeneous lesion. The lesion appeared T1 iso- to hyperintense and T2 hyperintense, involving the adductor muscles of the right thigh. It was in contact with the medial surface of the proximal shaft of the right femur and the inferior pubic ramus, with no obvious bony invasion noted. The adjacent neurovascular structures appeared normal, and a few vessels were seen surrounding the lesion (Figure 2).

**Figure 3:**
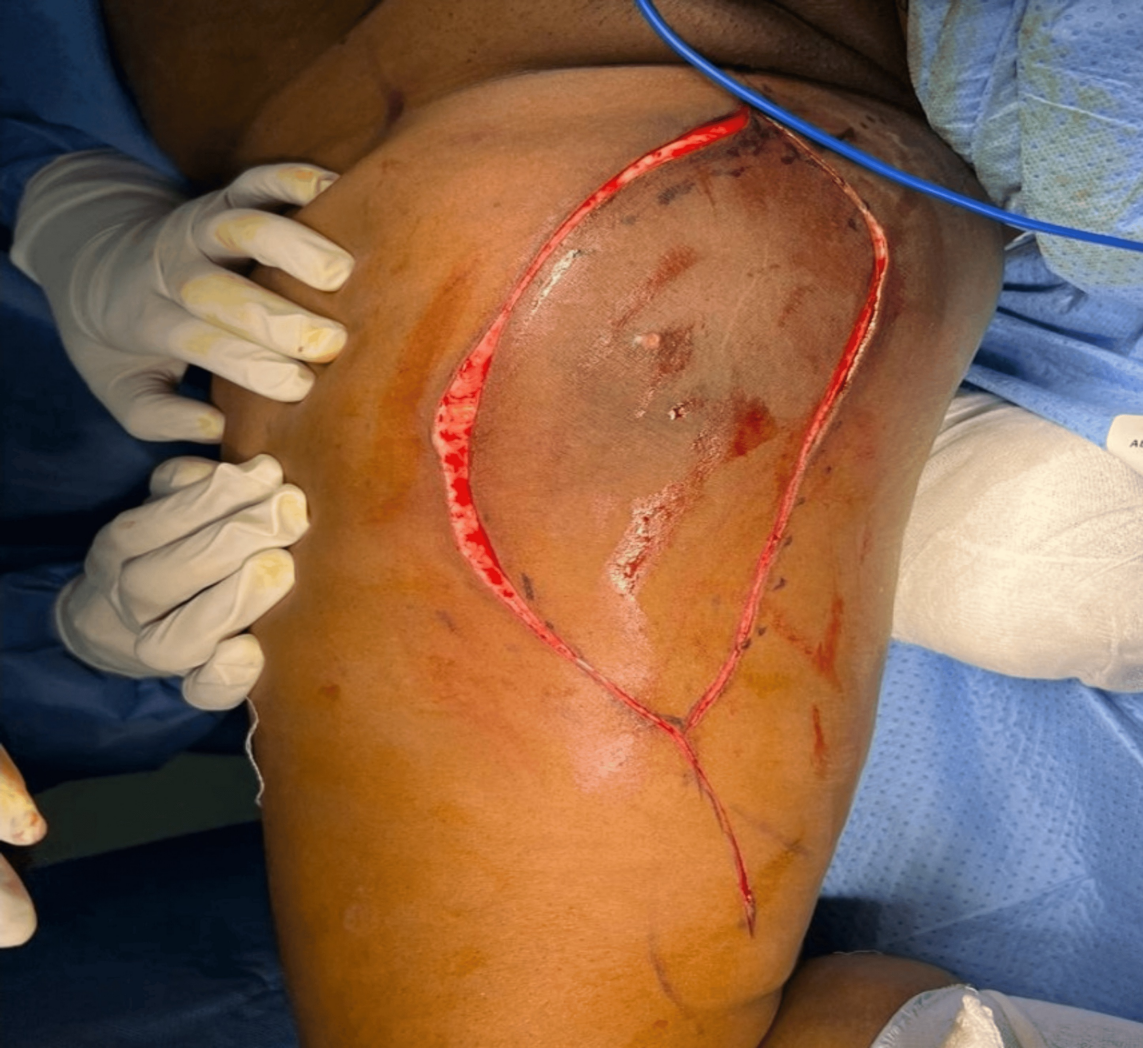
Vertical elliptical incision over the swelling.

A computed tomography (CT) scan of the chest, abdomen, and pelvis was conducted as part of the work-up, revealing no evidence of metastatic disease. Following a multidisciplinary team discussion regarding treatment options, the patient opted for a right medial thigh compartmental excision as a major elective procedure under general anaesthesia, with vertical elliptical incision deepened without raising fascia cutaneous flaps as shown in Figure 3.

**Figure 4:**
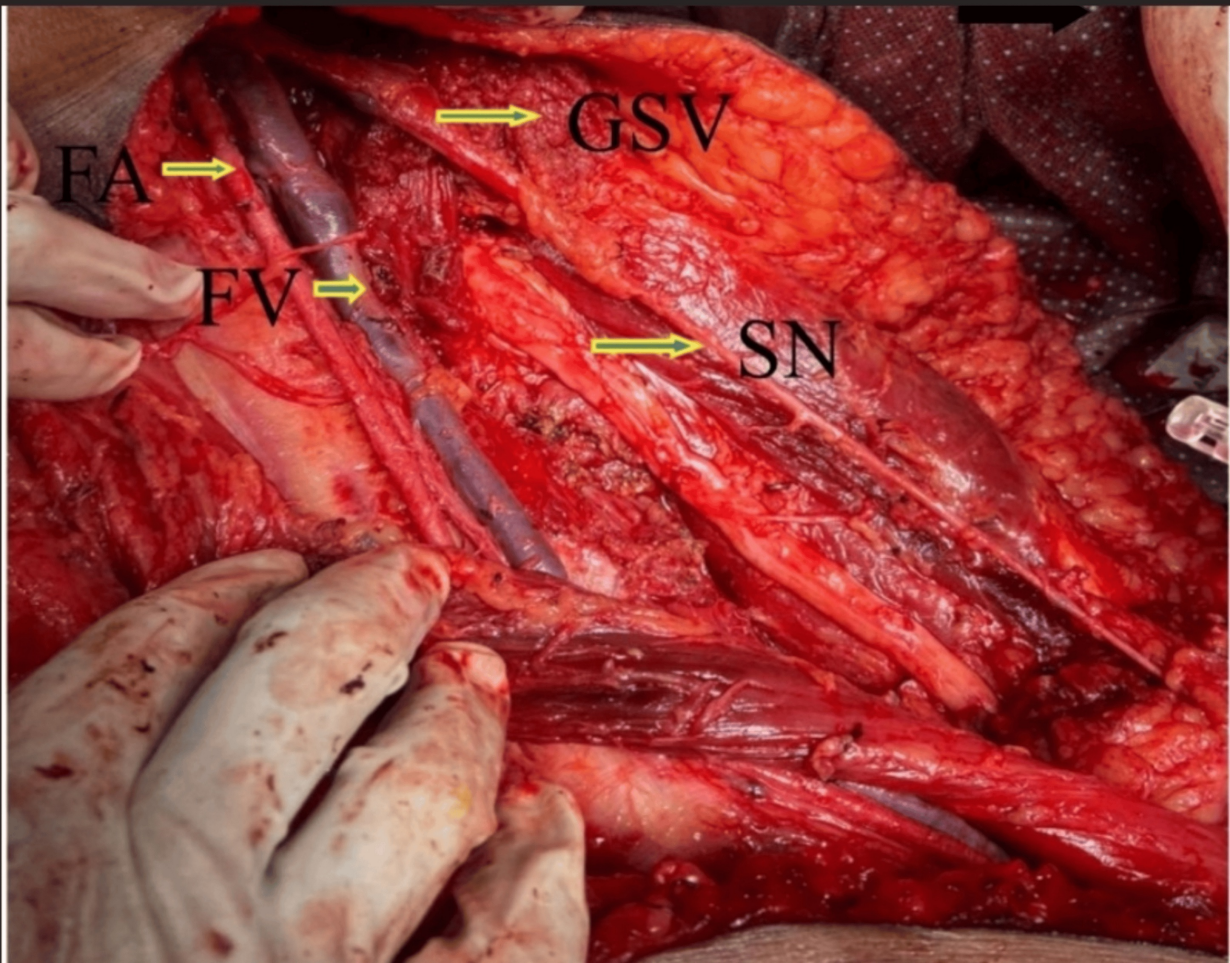
Tumor bed post excision of specimen. FA- Femoral artery; FV- Femoral vein; GSV- Great saphenous vein; SN- Sciatic nerve

**Figure 5:**
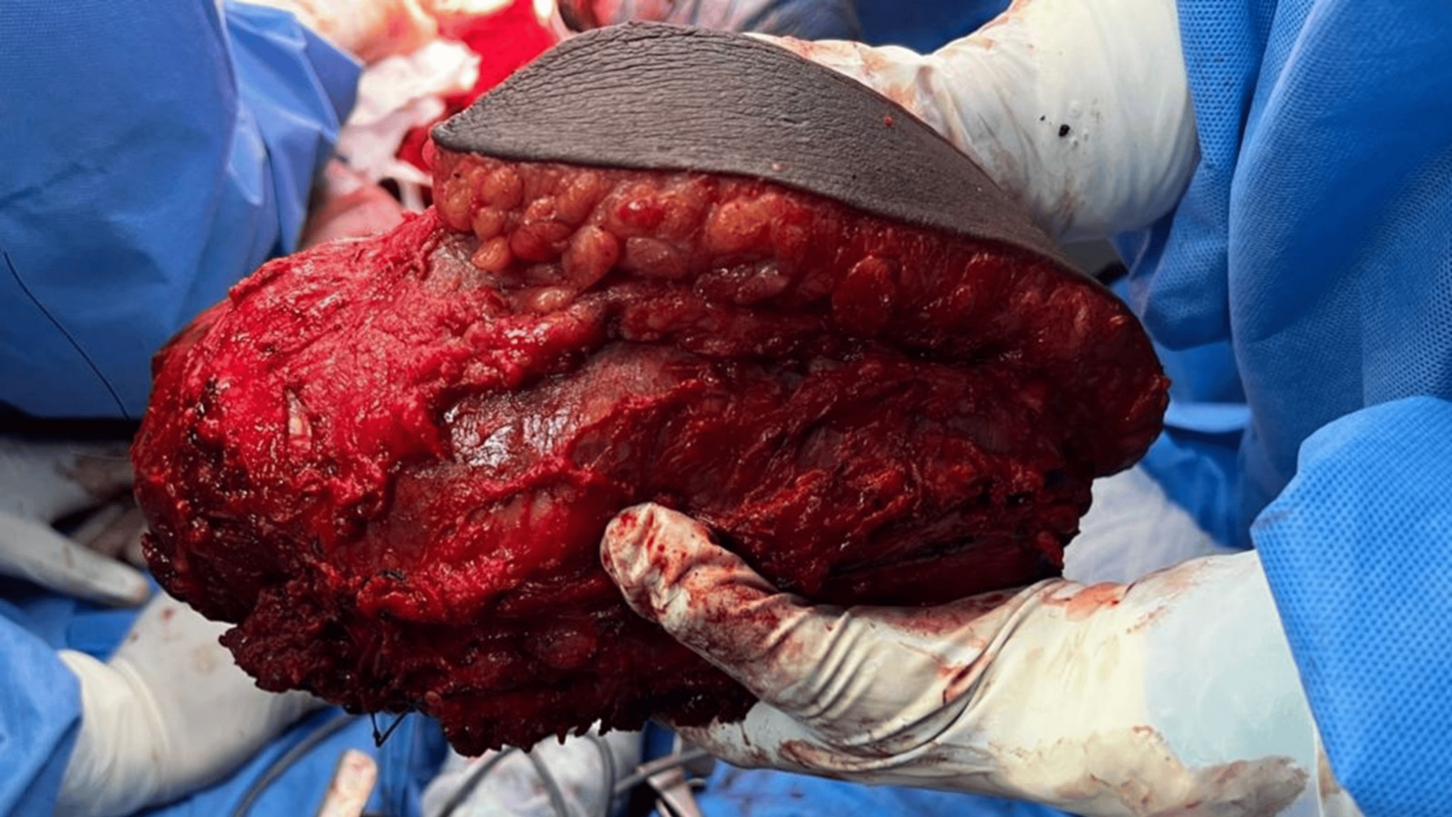
Gross image of the resected specimen with overlying fat and ellipse of skin.

Intraoperatively, a large tumor measuring 30 x 20 cm was found occupying the medial compartment. The vessels were splayed by the tumor, which abutted the medial surface of the femur, the pubic rami superiorly, and was in proximity to the femoral vessels. However, the great saphenous vein, femoral nerve, and sciatic nerve were all free of tumor involvement (Figure 4). The tumor was subsequently removed in toto, and the gross specimen was sent for histopathology (Figure 5).

**Figure 6:**
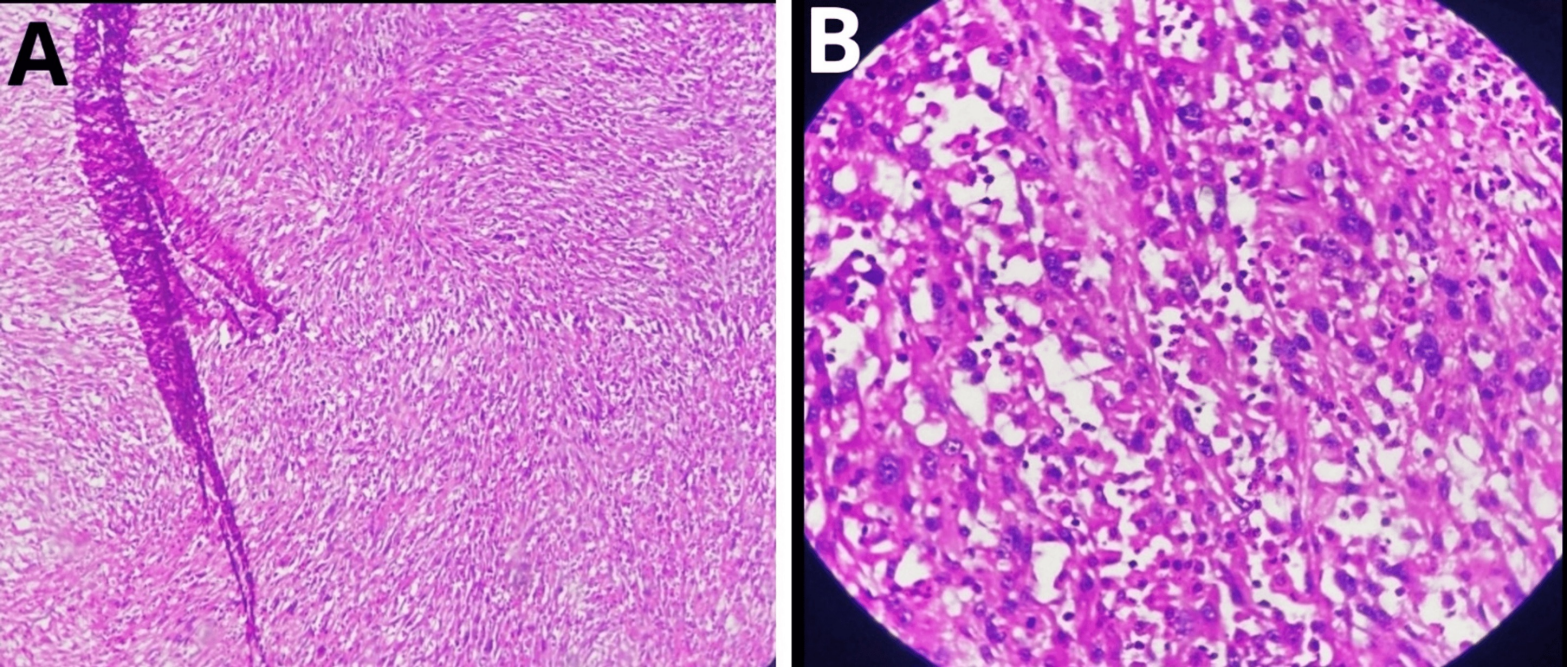
Histopathological features of tumor cell patterns in undifferentiated pleomorphic sarcoma (A) Histopathology image showing tumor cells arranged in a fascicular pattern, characterized by intersecting bundles of spindle-shaped cells. The cells display marked pleomorphism, with variable cell sizes and hyperchromatic nuclei, consistent with the features of a high-grade sarcoma. (B) Histopathology image showing small, round, blue tumor cells with hyperchromatic nuclei and scant cytoplasm. This pattern is indicative of a highly cellular tumor, with densely packed cells that appear uniform in size and shape. The histological appearance supports the diagnosis of an aggressive soft tissue sarcoma.

Histopathological examination revealed tumor cells exhibiting distinct patterns, including fascicular arrangements and clusters of small, round, blue tumor cells (Figure 6).

**Figure 7:**
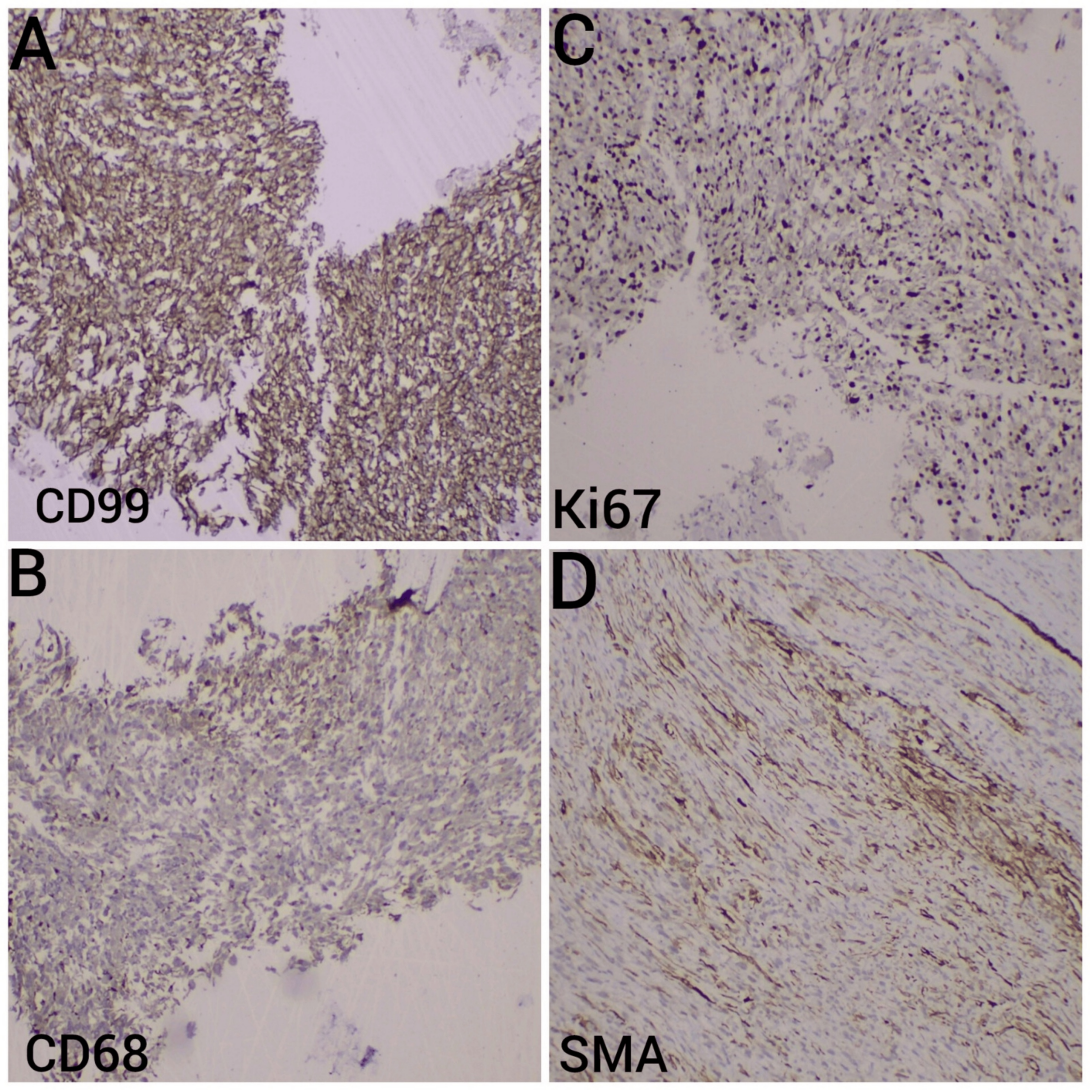
Immunohistochemical characterization of undifferentiated pleomorphic sarcoma Subimage (A): CD99 Staining. The tumor cells exhibit strong, diffuse cytoplasmic and membranous staining for CD99, demonstrating widespread expression throughout the tumor tissue. This staining supports the diagnosis of undifferentiated pleomorphic sarcoma, as CD99 is frequently linked to mesenchymal origin. Subimage (B): CD68 Staining. Immunohistochemistry for CD68 shows focal positivity in tumor cells, indicating areas of histiocytic differentiation. CD68, a marker of macrophages and histiocytes, aids in identifying the presence of macrophage-like features within the tumor, which is consistent with undifferentiated pleomorphic sarcoma. Subimage (C): Ki-67 Staining. The Ki-67 staining reveals a high proliferation index, with over 50% of tumor cell nuclei showing positive staining. This high level of Ki-67 expression indicates a highly proliferative tumor, confirming the high-grade nature of the undifferentiated pleomorphic sarcoma. Subimage (D): Smooth Muscle Actin (SMA) Staining. Occasional tumor cells demonstrate cytoplasmic positivity for smooth muscle actin (SMA), suggesting regions of smooth muscle differentiation. The presence of SMA-positive cells, although limited, underscores the heterogeneous nature of the tumor and supports the diagnosis.

Further immunohistochemical analysis confirmed the diagnosis of the tumor, highlighting its unique characteristics through CD99, CD68, Ki-67, and smooth muscle actin (SMA) staining (Figure 7).

This alignment of descriptions helps clarify the unique immunohistochemical characteristics of the tumor in each subimage, reinforcing the diagnosis and understanding of the tumor's aggressive behaviour and diverse cellular composition.

The tumor margins were close, with the nearest margin measuring 1 mm superomedially and superolaterally. Immunohistochemistry revealed positive staining for CD99, CD68, and occasional positive staining for smooth muscle actin (SMA). The tumor was negative for CD31, epithelial membrane antigen (EMA), CD30, TLE-1, desmin, S100, CD34, and H-CALDESMON. Ki-67 expression was greater than 50%, further supporting the diagnosis of undifferentiated pleomorphic sarcoma. Additionally, there was positive staining for CD10, while AE1/AE3, CD30, CD34, myoglobin, and desmin were negative.

The patient’s postoperative recovery was uneventful, with early mobilization and limb physiotherapy encouraged. She underwent a course of radiotherapy consisting of three cycles post-procedure. Follow-up MRI scans at six months and one year showed no evidence of recurrence.

## Discussion

Undifferentiated pleomorphic sarcomas (UPS) are rare, aggressive, high-grade tumors that lack a specific line of differentiation, making them diagnostically challenging [6]. These tumors predominantly arise in the lower extremities, followed by the retroperitoneum, arms, and head and neck [5, 8]. The typical demographic for UPS consists of patients in their seventh decade of life and beyond, making our case somewhat unusual due to the patient’s relatively younger age. Additionally, there was a preceding history of trauma, which, although not typically a causative factor, may have played a role in the discovery of the tumor.

The exact aetiology and pathogenesis of undifferentiated pleomorphic sarcomas remain undetermined [9]. Minor trauma triggering the development of soft tissue sarcomas is rare, and the association between these events is not well understood [7]. In this patient’s case, the association of trauma with the development of Undifferentiated Pleomorphic Sarcoma (UPS) remains challenging to establish definitively, though there are some theoretical and experimental underpinnings that suggest trauma might act as a promoter for sarcoma formation under certain conditions. While trauma may not directly cause sarcomas, it can draw attention to a previously unnoticed mass, as is often reported. In cases like ours, where a painful mass appears after minor trauma, it raises questions about whether the injury could have triggered or accelerated the growth of a pre-existing sarcoma, especially given the interval between the trauma and the noticeable increase in size of the mass [7].

Animal studies suggest a link between trauma, inflammation, and sarcoma formation, particularly in genetically predisposed tissues. For example, Rous sarcoma virus experiments in chickens showed tumor development at injury sites, and transgenic mice with oncogene activation also developed sarcomas at injury sites, supporting the idea of trauma as a promoter in tumorigenesis. In humans, proving a direct trauma-tumor link is complex, but trauma can lead to prolonged inflammation, which fosters tumor growth. For this patient, trauma may have promoted inflammation and cellular changes, potentially accelerating sarcoma formation in a genetically susceptible area, though it’s unclear if trauma caused or revealed a pre-existing lesion [7].

The primary presentation of UPS is often non-specific, with the majority of patients noticing a painless, enlarging mass [10]. This lack of distinctive symptoms can delay diagnosis, particularly in younger patients where malignancy may not be the immediate consideration. In our case, the patient presented with a painful mass, which is atypical and may have contributed to her seeking earlier medical attention.

The diagnostic work-up for soft tissue neoplasms typically begins with conventional radiographs, followed by magnetic resonance imaging (MRI) to assess the local extent of the tumor, its potential invasion into surrounding structures, and for preoperative planning. MRI remains the imaging modality of choice for soft tissue sarcomas due to its superior soft tissue resolution. Additionally, all patients diagnosed with soft tissue sarcoma should undergo a metastatic work-up, including high-resolution CT (HRCT) of the chest to evaluate for pulmonary metastases and a CT scan of the abdomen to assess for liver or retroperitoneal metastases [11]. In this case, no evidence of metastatic disease was found, allowing for a more focused local treatment plan.

The management of soft tissue sarcomas exemplifies the importance of a multidisciplinary approach to optimise outcomes, preserve limb function, and enhance the patient’s quality of life. Limb salvage procedures are particularly favoured in soft tissue sarcomas, where the goal is to achieve tumor control while maintaining as much functionality as possible [12].

Definitive treatment of UPS involves en bloc surgical excision, with an emphasis on achieving clear microscopic margins to reduce the risk of local recurrence. Due to the non-specific nature of UPS, making a definitive pathological diagnosis requires multiple immunohistochemical stains, as it is largely a diagnosis of exclusion [13]. In our patient, immunohistochemistry confirmed the diagnosis of UPS, and given the presence of positive postoperative margins, she was subsequently treated with postoperative radiotherapy to reduce the likelihood of local recurrence.

The role of adjuvant chemotherapy in the treatment of resectable soft tissue sarcomas remains controversial [14]. While some studies suggest a benefit in terms of recurrence and survival, the evidence is not conclusive, and its use is typically determined on a case-by-case basis. In this instance, adjuvant chemotherapy was not administered, though the patient will remain under close surveillance.

UPS carries a significant risk of recurrence, even following complete resection. Factors associated with a higher risk of recurrence include tumor size greater than 5 cm, deep-seated tumors, and high-grade histology. The recurrence rate in such cases can reach 40-50% [14, 15]. Given this high risk, patients require stringent surveillance, which includes physical examinations and radiographic imaging every three to six months for the first two to three years postoperatively. After this period, the frequency of surveillance can be reduced to every six months for the subsequent two years and annually thereafter [16].

The limitations of this case report include the difficulty in establishing a direct causal relationship between trauma and the development of Undifferentiated Pleomorphic Sarcoma (UPS), as no clear genetic or predisposing factors were identified in the patient. Lastly, conclusions drawn from animal studies on trauma and sarcoma formation may not fully apply to humans due to biological differences, limiting the generalizability of these findings.

## Conclusions

Although any soft tissue swelling greater than 5 cm should raise suspicion for a soft tissue sarcoma, these tumors are often overlooked due to their rarity and non-specific clinical presentation. Their vague symptoms may be mistakenly attributed to benign causes, leading to delays in diagnosis. Consequently, the presence of any large soft tissue mass should trigger a high degree of suspicion for malignancy, with undifferentiated pleomorphic sarcoma as a potential diagnosis. This case underscores the importance of recognizing the specific features of soft tissue sarcoma, particularly when it follows trauma. While the link between trauma and sarcoma development is not well established, clinicians should remain vigilant, as early detection is crucial. Timely diagnosis followed by complete surgical excision of undifferentiated pleomorphic sarcomas is essential for improving oncological outcomes and ensuring the best possible prognosis for the patient.
